# Coagulase-Negative Staphylococci Pathogenomics

**DOI:** 10.3390/ijms20051215

**Published:** 2019-03-11

**Authors:** Xavier Argemi, Yves Hansmann, Kevin Prola, Gilles Prévost

**Affiliations:** 1 Service des Maladies Infectieuses et Tropicales, Hôpitaux Universitaires, 67000 Strasbourg, France; yves.hansmann@chru-strasbourg.fr; 2 Université de Strasbourg, CHRU Strasbourg, Fédération de Médecine Translationnelle de Strasbourg, EA 7290, Virulence Bactérienne Précoce, F-67000 Strasbourg, France; kevin.prola@etu.unistra.fr (K.P.); prevost@unistra.fr (G.P.)

**Keywords:** pathogenomics, coagulase-negative staphylococci, virulence factors, whole genome sequencing

## Abstract

Coagulase-negative Staphylococci (CoNS) are skin commensal bacteria. Besides their role in maintaining homeostasis, CoNS have emerged as major pathogens in nosocomial settings. Several studies have investigated the molecular basis for this emergence and identified multiple putative virulence factors with regards to *Staphylococcus aureus* pathogenicity. In the last decade, numerous CoNS whole-genome sequences have been released, leading to the identification of numerous putative virulence factors. Koch’s postulates and the molecular rendition of these postulates, established by Stanley Falkow in 1988, do not explain the microbial pathogenicity of CoNS. However, whole-genome sequence data has shed new light on CoNS pathogenicity. In this review, we analyzed the contribution of genomics in defining CoNS virulence, focusing on the most frequent and pathogenic CoNS species: *S. epidermidis*, *S. haemolyticus, S. saprophyticus*, *S. capitis*, and *S. lugdunensis*.

## 1. Introduction

Bacterial virulence is a complex concept that must be considered from the clinical, molecular, and genomic perspectives. Clinically, the virulence of a pathogen, of a specific species or even of a clonal strain, can refer to its inherent capacity to provoke specific clinical manifestations that can be linked to the production of a virulence factor, often a protein. Microbiologically, Robert Koch postulates illustrated bacterial virulence, which Stanley Falkow redefined in the late 1980s to provide a molecular version of it [1]. Molecularly, the virulence factor is found in the pathogenic strains of a species and not in non-pathogenic ones. Secondly, the inactivation of the encoding gene in an animal model should attenuate the virulence of the strain. Thirdly, the reintroduction/reactivation of the gene should restore virulence. Nevertheless, in the past 20 years, neither Koch’s nor Falkow’s hypotheses have been able to completely define virulence factors. The development of rapid and cost-effective genome sequencing technologies has provided the opportunity, among others, to discover a wide range of genes that could be linked to bacterial pathogenicity. This ability to determine the pathogenic capacities of a bacterium from its genome is known as pathogenomics [2]. Virulence factor databases allow for fast and easy identification of putative virulence factors in whole-genome sequence data based only on sequence homologies. The Virulence Factor Database was released in 2005 and is an example of a regularly-updated database, of which a fourth version was released in 2018 [3]. Other updated databases exist, such as PHI-base and Victors [4,5]. Thus, some virulence factors retain the definition of Stanley Falkow, with a direct causative link with bacterial pathogenicity and some possible clinical manifestations. Conversely, some other factors, such as those that are produced by human commensal bacteria, do not act as virulence factors under normal conditions, but these factors will take part in bacterial pathogenicity under specific conditions, such as during cutaneous barrier breach. Staphylococci are examples of such dual characterizations of virulence factors.

Staphylococci comprise *Staphylococcus aureus*, which is a human commensal bacterium that colonizes nearly 30% of the human skin and mucosae, and coagulase-negative staphylococci (CoNS), which include species such as *Staphylococcus epidermidis*, which colonizes nearly all human skin [6]. *S. aureus* is usually considered separately from CoNS and as a virulent species. Indeed, it produces a wide range of toxins which can act as virulence factors leading to specific clinical manifestations with a direct causative link. For example, the Panton-Valentine leucocidin (LukSF-PV) is a bicomponent toxin that may lead to necrotizing pneumonia [7]. *S. aureus* produces other toxins such as epidermolysins (ETA-B-D) and superantigens, e.g., toxic shock syndrome toxin (TSST-1), that lead to specific clinical diseases and turn some particular strains into pathogens that fully meet the Koch and Falkow postulates [8,9]. *S. aureus* might also produce numerous adhesion factors, capsule, and biofilm-associated proteins, hemolysins, and immune evasion proteins whose main purpose is to persist on human skin as a commensal and probably beneficial bacterium [10]. Many of those proteins are cell wall-anchored and comprise a group of covalently-attached proteins with specific motifs that bind to the extracellular matrix and are usually identified as microbial surface components recognizing adhesive matrix molecules (MSCRAMMs) [11]. Many other proteins are cell-wall-attached but display distinct functions such as iron-regulated surface proteins, and some genes encode for putative MSCRAMMs but without any molecular and structural confirmation, and can be identified more generally as surface adhesins, a terminology used in this review. Those adhesion factors play a crucial role in the pathogenicity *of S. aureus*. The occurrence of a cutaneous barrier breach will allow its penetration in a new environment, where those factors will behave as real virulence factors, allowing its adherence, persistence, and multiplication in a normally sterile environment. Whole-genome sequence analysis of *S. aureus* has revealed the existence of a very large repertoire of such toxins, adhesion molecules, and biofilm-associated genes, sometimes without any molecular confirmation but based on sequence homologies that lead to the characterization of *S. aureus* as a major pathogenic species [12].

CoNS are often considered as simple commensal bacteria due to their rarity in clinical pathology and the absence of virulence factors like those produced by *S. aureus.* However, the emergence of nosocomial infections with CoNS has led clinicians and researchers to reconsider the status of these bacteria [13]. The publication of several CoNS genomes has been an essential tool to better understand CoNS pathogenicity. The identification of virulence factors, such as toxins with clinical impacts, remains exceptional or even controversial, and it appears that CoNS have a very large repertoire of genes that encode for adhesion factors, biofilm production, hemolysins, exoenzymes, and superantigens. Most CoNS also have similar regulatory systems to *S. aureus*, e.g., the *agr* system, for which homologs have been identified in numerous CoNS species [13]. The cutaneous barrier breach is a critical step to turning the CoNS species into pathogens, turning factors that are mainly implicated in the bacterial life cycle on the skin into virulence factors leading to pathological manifestations. In this situation, once again, the concept of virulence factors does not apply to the definitions provided by Robert Koch and Stanley Falkow. Considering the great species diversity of CoNS, *S. epidermidis* is the most frequent CoNS implicated in clinical diseases, and it remains the best-studied species at a molecular and genomic level [14]. Nevertheless, none of the virulence factors that were discovered could clearly categorized a specific strain as pathogenic or commensal, even if the strains belonging to the clonal type ST2 have been over-represented in the specific context of *S. epidermidis* neonatal sepsis [15]. ST2 clonal type always bears the insertion sequence *IS256* and *ica* genes, which are implicated in biofilm production, but the direct correlation of those loci with *S. epidermidis* invasiveness has never been confirmed [16]. Some other species also came up as significant pathogens, such as *S. lugdunensis* that has been widely investigated due to the severity and the specificity of clinical manifestations of infection due to this species [17]. *S. haemolyticus*, *S. saprophyticus,* and *S. capitis* are three other species for which the rate and the type of infection led to molecular and genomic investigations in the search of virulence factors [18,19]. *S. caprae* is a CoNS that usually belongs to the animal skin flora, but it can also colonize human skin. This CoNS has been described in veterinary infections (mastitis principally) and in human osteoarticular infections. Interestingly, this CoNS belongs to a group of CoNS that is comprised of *S. epidermidis*, *S. capitis*, and *S. saccharolyticus* [13,20,21,22].

The identification of virulence factors in staphylococci has resulted in the identification of mobile genetic elements. Indeed, the majority of *S. aureus* virulence genes are located on mobile genetic elements such as pathogenicity islands, plasmids, and phages, which represent up to 25% of the *S. aureus* genome and play crucial roles in host adaptation and the modulation of virulence [23,24,25]. Several bioinformatic tools have been developed to identify such genomic regions based on comparative genomics [26,27,28]. Thus, the identification of putative virulence factors can be driven by the identification of these genomic elements, but it has appeared that the presence of such mobile genetic elements is very common in CoNS, but that they do not bear any virulence factors which are similar to *S. aureus.* More generally, this large repertoire of mobile genetic elements in staphylococci species explains why, despite a limited core genome, those species usually displayed an open pan genome with a very high number of existing genes, sometimes shared by a very limited number of clones [29,30,31]. Worthy of note is the fact that *S. lugdunensis* has a closed pan genome which contains several barriers to horizontal gene transfers, which could explain this unique genomic characteristic amongst CoNS [32].

In this review, we aimed to describe the occurrence of virulence factors, or more accurately, of pathogenicity factors, based on whole-genome sequence data. We focused this systematic review on the major CoNS pathogens for which such data have been published: *S. epidermidis*, *S. lugdunensis*, *S. saprophyticus, S. haemolyticus*, *S. caprae*, and *S. capitis.* A comprehensive review of all CoNS pathogenicity factors has been described elsewhere and is covered in references [13,33,34,35,36,37,38,39].

## 2. Research Method and Results

This systematic review was based on PubMed published articles in the English language. We used the following MeSH terms: “Virulence”, “Virulence factors”, “Pathogenicity”, “Whole genome sequencing”, and “Genomics”. In particular, we searched for the following species: “*Staphylococcus epidermidis*”, “*Staphylococcus lugdunensis*”, “*Staphylococcus capitis*”, “*Staphylococcus caprae*”, “*Staphylococcus haemolyticus*”, and “*Staphylococcus saprophyticus*”. We excluded studies that were not based on whole-genome data, and those for which genome sequences were not deposited in public databases (e.g., GenBank, European Nucleotide Archive).

We identified eight studies for *S. epidermidis* [29,30,40,41,42,43,44,45], four for *S. capitis* [46,47,48,49], three for *S. lugdunensis* [32,50,51], two for *S. haemolyticus* [52,53], and one for *S. caprae* [54] and *S. saprophyticus* [55].

### 2.1. Staphylococcus epidermidis

*S. epidermidis* is by far the most prevalent CoNS in microbiological samples and the primary cause of CoNS-related infections, particularly in nosocomial settings [56]. This species has been widely studied at a molecular level and has provided the largest amount of data regarding the presence of virulence factors that might be identified as pathogenicity factors [16]. Biofilm formation is the main path by which *S. epidermidis* colonizes and infects prosthetic and medical devices as reviewed by Otto et al. [57]. The biofilm life cycle is a complex process which implicates MSCRAMMs, the proliferation of the exopolysaccharide matrix, and finally, biofilm dispersion, mostly due to phenol-soluble modulin (PSM) peptides [57].

Nearly 500 genomes have been made available on GenBank, mainly draft genomes, but also 12 complete genomes. *S. epidermidis* whole-genome-based studies to determine virulence determinants are detailed in Table 1. In 2003, Zhang et al. provided the first genome-based analysis of *S. epidermidis* virulence [40]. The authors provided the first sequence of the reference strain ATCC 12228, a non-biofilm forming, non-infection associated strain, and they found several putative pathogenicity factors, including multiple MSCRAMMs encoding genes and putative exoenzymes and toxins such as metalloproteases and Delta/Beta-hemolysins. Interestingly, a revised version of this genome was released in 2017 which was determined using PacBio (PacBio, Pacific Biosciences, Menlo Park, CA, USA) long read sequencing; it provided a slightly different genome from the one released by Zhang et al., and established a new reference genome for comparative genomic studies [58]. In 2005, Gill et al. published a new complete genome from *S. epidermidis* strain RP62a and performed comparative genomic analyses with four *S. aureus* genomes, and included the genome from *S. epidermidis* ATCC 12228 [41]. As expected, the authors found multiple virulence factors in the *S. aureus* genome. Nearly one-half of those factors were carried by seven pathogenicity islands that were not found on *S. epidermidis*. Nevertheless, some other mobile genetic elements were identified in this species (one genomic island and two integrated plasmids), some bearing PSM and putative MSCRAMMs. Some other mobile genetic elements were found in *S. epidermidis* genomes such as prophages, insertion sequences, and *SSCmec-like* cassettes. The authors also identified several other putative virulence factors such as proteases (serine and cysteine protease), lipases, and hemolysins (*Beta/Delta haemolysin*) loci. The presence of mobile genetic elements and virulence factors led the authors to consider that horizontal gene transfer between staphylococci had to be considered as a source of variability and, in this case, as a cause of their pathogenicity.

Conlan et al. provided in 2012 the first large comparative genomic analysis of 30 *S. epidermidis* whole genomes, showing that *S. epidermidis* had an open pan genome, as expected, but more interestingly, that whole-genome-based phylogenetic trees could distinguish between commensal and pathogenic strains. The presence of the *formate dehydrogenase* gene (*fdh*) could explain this observation [42]. Meric et al. found in a second study based on whole-genome comparative analyses that *S. epidermidis* and *S. aureus*, in hospital settings, share some genes involved in pathogenicity, suggesting the existence of horizontal gene transfer between these species [29]. The added value of whole-genome analysis was confirmed by Virginia Post et al., who compared whole-genome sequences of 104 *S. epidermidis* isolates from patients with orthopedic-device-related infections. This study found a correlation between patient outcome and some loci [30]. Patients with multiple surgeries due to treatment failure were more likely to be infected with the biofilm-associated gene *bhp*, the antiseptic resistance gene *qacA*, the cassette genes *ccrA* and *ccrB*, and the *IS256-like* transposase gene. This whole-genome-based study identified, for the first time and directly from whole-genome data, loci that might be involved in *S. epidermidis* pathogenicity based on well-documented clinical data.

While such studies have yielded novel and sometimes revolutionary insights into *S. epidermidis* virulence, the presence of such loci does not provide any information regarding their expression, regulation, and their impact on clinical implications. Yet, whole-genome data can be used as a complementary tool when a putative virulence factor is identified, sometimes starting from clinical considerations. In 2013, Fournier et al. provided observations of a patient with *S. epidermidis* endocarditis, and the authors found several clues that could explain the virulence of this strain [43]. Besides known virulence factors such as MSCRAMMs, exoenzymes, and hemolysins, the authors identified a previously unreported prophage, a new toxin/antitoxin module, and a complete *icaABCD* operon, which was usually not observed in non-pathogenic strains. As observed by Conlan et al., this pathogenic strain also lacked the *fdh* gene.

The existence of enterotoxins and pathogenicity islands in *S. epidermidis* has been controversial, despite observations by Madhusoodanan et al. of such a genetic element in the clinical strain *S. epidermidis* FRI909, which carried two functional enterotoxin genes *sec3* and *sell* [59]. This unique observation was initially considered as a possible genetic “accident”. In 2016, we described two strains isolated from septic shock patients that produced a staphylococcal enterotoxin C with homology to *S. aureus* enterotoxin C3 [60]. By using whole-genome data, we thereafter provided evidence that the *sec3* from our strains was located on a pathogenicity island very similar to the one described by Madhusoodanan et al., named SePI-1/SeCI-1 [44]. We also identified several plasmids carrying resistance genes and sharing homologies with *S. aureus*. This result confirmed that some mobile genetic elements from *S. epidermidis* might come from *S. aureus*, with the transfer of virulence factors.

Recently, Xu et al. performed a whole-genome analysis of a multi-resistant *S. epidermidis* strain isolated in the environment, i.e., in a hotel room, and identified multiple loci dedicated to antibiotic resistance but also to numerous virulence genes, as described previously [45].

*S. epidermidis* is a major cause of nosocomial infections within other CoNS. The existence of several factors that modulate its commensal life cycle on the skin also turns this species into a pathogen when entering sterile sites. Whole-genome studies have provided specific and unique data regarding the general evolution of its genome and interactions with other staphylococci, such as *S. aureus*. However, whole-genome data also appear as crucial and unique complementary analyses when a specific strain is isolated clinically, or when a putative virulence factor is molecularly identified.

### 2.2. Staphylococcus lugdunensis

*S. lugdunensis* is considered a significant pathogen in human infection [17]. Clinically, its virulence is most likely lower than that of *S. aureus*. Clinical observations have emphasized the frequency and severity of skin and soft tissue infections, endocarditis, and osteoarticular infections [61,62,63]. Microbiologically, this CoNS produces a coagulase, which is a common feature with *S. aureus* [64]. Its pathogenicity has been noted by several authors, and multiple virulence factors have been identified molecularly, including adhesion molecules like a fibrinogen-binding protein; cytotoxins such as δ-like-hemolysin; a complete biofilm synthesis system, including an *agr* regulating operon; and as seen in *S. aureus*, a large system dedicated to iron metabolism [65,66,67]. Interestingly, Heilbronner et al. showed that duplication of the *isd* locus provided a selective advantage to *S. lugdunensis* in the case of iron limitation, possibly improving bacteria survival and pathogenicity. Recently, we identified and purified a novel metalloprotease named lugdulysin that could be implicated in osteoarticular infections [61].

*S. lugdunensis* whole-genome-based studies to determine virulence determinants are listed in Table 2. The first complete genome of *S. lugdunensis* was published by Tse et al. in 2010 [68]. Since then, 17 complete genomes have been published, and eight additional draft genomes are available in GenBank. Nevertheless, the first exploitation of a complete genome was provided by Heilbronner et al. in 2011 [50]. The authors published the complete genome of strain N920143 and identified several putative virulence factors based on comparative genomics and homologies searches. They identified, for the first time, a complete operon implicated in iron metabolism (*isd*: *isdB-J-C-K-E-F-G*), several genes coding for MSCRAMMs such as fibrinogen and Von Willebrand adhesion factors, a *streptolysin S-like toxin*, an *IcaABCD* operon for biofilm synthesis, *agr* regulation genes, and even an *esx* locus with homology to the esx-ESAT 6 secretion systems that are implicated in the secretion of virulence factors in *Mycobacterium tuberculosis*. Due to the frequent localization of virulence genes on mobile genetic elements in *S. aureus*, in 2017 we also published the complete genome of seven strains of *S. lugdunensis* and identified multiple mobile genetic elements [51]. We identified a very unusual number of plasmids and prophages by using computational methods, including four new prophages and five plasmids, in which three were previously described in other CoNS and one in *S. aureus*. This study suggested that horizontal gene exchange could occur between staphylococci, including *S. aureus*. Descriptions of plasmids and prophages in CoNS remain rare, but, interestingly, they have been performed mainly in *S. epidermidis* and *S. haemolyticus*.

*S. lugdunensis* is characterized by its unusual antimicrobial susceptibility, even if some rare strains are oxacillin-resistant. In 2017, Chang et al. fully characterized, for the first time, two novel variants of staphylococcal cassette chromosome *mec* elements in two oxacillin-resistant *S. lugdunensis* strains by using whole-genome sequence data [69]. The authors also identified several homologies with *S. aureus*, *S. haemolyticus*, and *S. epidermidis* cassette chromosome regions. If this result suggested once again the existence of horizontal gene exchanges between staphylococci, including *S. aureus*, the very unusual antimicrobial susceptibly of *S. lugdunensis* remained unexplained in comparison to other CoNS, which displayed a very high rate of resistance, particularly in nosocomial settings [19]. We recently published a comparative genomic analysis of *S. lugdunensis* whole-genome with *S. aureus* and *S. epidermidis* [32]. *S. epidermidis* and *S. aureus* are characterized by an open pan genome, which means that those species display a virtually unlimited number of new genes when adding different genomes, even if they conserve a limited number of genes through the strains called the core genome. Conversely, *S. lugdunensis* display a closed pan genome, and all published genomes were very similar, with only a very limited number of new genes among the strains. This unexpected characteristic could explain the very well conserved antimicrobial susceptibility of *S. lugdunensis* that rarely acquires resistance genes. We also found that this observation could rely on the presence of numerous barriers to horizontal gene transfer in this species, including restriction-modification, CRISPR/Cas, and toxin/antitoxin systems.

*S. lugdunensis* is a very particular CoNS and might be closer to *S. aureus* than other CoNS in terms of virulence. Whole-genome sequence studies have emphasized its originality on a genomic scale, including the presence of multiple unusual factors implicated in its pathogenicity and that are uncommon for CoNS, like its iron metabolism system. Overall, its genome encodes for several factors that take part in its survival on the skin as a commensal bacterium, such as adhesion proteins and biofilm capacities. Once again, those factors probably act as virulence factors once the cutaneous barrier is broken. Comparative genomic studies have also revealed that it displays a unique profile in terms of genomic plasticity, which could explain some of its microbiological characteristics, such as its highly-conserved antimicrobial susceptibility.

### 2.3. Staphylococcus capitis

*S. capitis* is the third CoNS that has been described in clinical infections and for which whole-genome studies focusing on its virulence are available [13]. *S. capitis* infections have been described in various clinical situations implicating biofilm production as endocarditis, catheter-related bacteremia, and prosthetic joint infections [70,71]. This species also causes neonatal sepsis in neonatal intensive care units [72,73].

*S. capitis* whole-genome-based studies to determine virulence determinants are listed in Table 3. The first draft genome of this species was released in 2009, and the first complete genome was published in 2015 by Cameron et al. to determine the genetic determinants that could support its pathogenicity [46]. The authors identified several loci that could encode for virulence factors and performed a comparative genomic analysis with the *S. epidermidis* genome. They identified putative virulence regulators (as *agr* homologs), biofilm production loci, genes encoding for exoenzymes (as metalloproteases and hemolysins), PSMs, and MSCRAMMs. This genome-scale analysis led to a functional analysis of biofilm and PSMs production.

At a larger scale, it appears that some clonal strains might be implicated in the context of neonatal sepsis as suggested by Butin et al., who observed the worldwide endemicity in 17 countries of a multidrug-resistant strain by using pulsed-field gel electrophoresis patterns but also whole-genome sequence data [74]. Multidrug resistance has emerged as a crucial issue in *S. capitis* infections, with nearly half of the strains being oxacillin-resistant in hospital settings; occasionally, some strains with vancomycin reduced susceptibility have been identified [72,73]. Li et al. provided a complete whole-genome analysis of a multi-resistant strain causing bacteremia, and found a very high and unusual number of genes implicated in such a resistant profile [47]. Recently, Simoes et al. published the first whole-genome sequence of *S. capitis* strain NRCS-A, which is a multi-resistant clone implicated in neonatal infections worldwide, by using PacBio (PacBio, Pacific Biosciences, Menlo Park, CA, USA) long read sequencing, and provided comparative genomic analysis with three other NRCS-A sequenced clones coming from different countries [48]. The authors identified, as expected, multiple resistant genes, but interestingly, some of them were located on mobile genetic elements. The authors identified several virulence genes which had been described previously by Cameron et al., but more specifically, they identified which of them was clone-specific as a putative restriction-modification system and a nisin resistance gene, which could explain the persistence of NRCS-A clones in patients’ gut microbiota. In 2017, Kumar et al. used whole-genome sequence data to reveal the existence of four loci encoding for antimicrobial peptides [49]. The authors were then able to perform a functional analysis to confirm the functionality (antimicrobial activity against Gram-positive bacteria, including *S. aureus*) of those genes, and formulate the hypothesis that such antimicrobial peptides could explain the adaptation and the persistence of *S. capitis* on the human skin.

*S. capitis* is considered a serious pathogen, particularly in neonatal settings, and whole-genome studies have provided useful and unique data regarding the spread of its antimicrobial resistance, but have also emphasized its ability to colonize and persist on human skin, which is directly linked to its pathogenicity regarding catheter-associated infections. 

### 2.4. Staphylococcus caprae

*S. caprae* is a normal component of the animal skin flora and can cause mastitis, principally in goats [13]. It also belongs to the human skin flora and is an observed cause of osteoarticular infections and also material-associated infections and bacteremia [20]. Until recently, only six draft genomes of *S. caprae* were available from humans and goats. *S. caprae* whole-genome-based studies to determine virulence determinants are listed in Table 4. In 2018, Watanabe et al. published a comparative genomic analysis of three human strains by performing a whole-genome assembly which leads to three complete chromosomes [54]. The authors compared the conserved genome parts of *S. epidermidis*, *S. capitis*, and *S. caprae*, as all previous phylogeny studies found all three species belonged to the same group (*S. epidermidis* group) [22]. They found that all three species shared a biofilm- and capsule-associated loci, and they also shared PSM genes. They also identified several putative MSCRAMMs genes. We observed that the three species belonged to the *S. epidermidis* group and shared very common features for skin resident bacteria. The similarity in clinical presentation of infection caused by these species in human disease has been illuminated.

### 2.5. Staphylococcus haemolyticus

*S. haemolyticus* is a commensal bacterium but is also a frequent nosocomial pathogen that has been described mainly in catheter-related bacteremia, with one of the highest degrees of methicillin resistance among CoNS [19,75,76]. *S. haemolyticus* whole-genome-based studies to determine virulence determinants are listed in Table 4. *S. haemolyticus* was one of the first CoNS with a complete genome published by Takeuchi et al. in 2005 [52]. The authors identified several putative virulence factors in this pioneering study, such as hemolysins and bacterial capsule. Until now, no publication has completely explored the presence of virulence genes in this species, warranting future studies. Some previous studies have described the presence of cytotoxins, enterotoxins, and PSMs at the molecular level, which is exceptional among CoNS [77,78]. This species is also a biofilm-producing species, but interestingly, in 2016, Hong et al. published the first complete sequence of *S. haemolyticus* by using PacBio (PacBio, Pacific Biosciences, Menlo Park, CA, USA) long read sequencing and found no *ica* operon, but an *agr* regulation system homolog, suggesting that alternative mechanisms were implicated in biofilm formation [53]. Thus, *S. haemolyticus* is a significant pathogen in humans, but it appears that a complete whole-genome analysis of those virulence factors is lacking, whereas, several complete genomes are already available with this species bearing very unusual virulence factors for a CoNS.

### 2.6. Staphylococcus saprophyticus

*S. saprophyticus* is a commensal CoNS and can lead to lower urinary tract infections in young women [79,80]. Colonization of the genital area and the gut has been linked to this atypical type of infection, but most importantly, it was shown in vitro that this CoNS has an unusual capacity to adhere to urothelial cells and to produce urease. Nevertheless, those characteristics are not unique in CoNS and do not fully explain the capacity of this CoNS to cause urinary tract infections. *S. saprophyticus* whole-genome-based studies to determine virulence determinants are listed in Table 4. In 2005, Kuroda et al. established the first complete genome of *S. saprophyticus*, and until now, only six complete genomes for this species have been made available in GenBank [55]. The authors identified three factors that could explain the specificity of *S. saprophyticus* pathogenicity by using whole-genome data and a comparative genomic approach. They identified a novel—and unique among CoNS—cell wall-anchored protein, UafA, associated at a molecular level with a high capacity to adhere to cells from the urinary tract. They also identified a unique uro-adaptative transport system and urease production as the two other factors that could be linked to the specific pathogenicity of *S. saprophyticus*. Since 2005, whole-genome virulence studies have been lacking, but adherence and persistence onto the urinary tract have been confirmed as the main factors that could be linked to pathogenicity [81,82,83]. Overall, *S. saprophyticus*, lacks many of the adhesion proteins and other virulence factors that have been identified in CoNS from the *S. epidermidis* group, *S. caprae*, and *S. lugdunensis*, which probably explains the differences that are observed at a clinical level. 

### 2.7. Phylogenetic Relationship among Staphylococcus Species

In 2012, Lamers et al. proposed a refined classification of staphylococci using a combination of Bayesian and maximum likelihood analysis of multilocus data based on four loci (non coding 16S rRNA, *dnaJ*, *rpoB*, and *tuf*) [22]. The authors identified six species groups and 15 cluster groups among staphylococci, a classification that is considered as a current standard for staphylococci phylogenetic classification [13]. Interestingly, *S. lugdunensis* appeared in a unique cluster group, a particularity that could be link to the very low allelic polymorphism of this species and the existence of a closed pangenome, a unique characteristic among CoNS [32,84]. In addition, *S. epidermidis*, *S. capare*, and *S. capitis* which are described here as pathogenic species in nosocomial settings belong to the same cluster group, along with *S. saccharolyticus*. *S. saprophyticus* and *S. haemolyticus* belong to distinct cluster groups in this classification. This classification does not clarify the mechanisms by which staphylococci acquire genes, including virulence factors, but only emphasize the uniqueness of *S. lugdunensis*, even if more generally, all staphylococci have strong barriers preventing horizontal gene transfers, making their transformation extremely difficult even in vitro [85].

## 3. Conclusions

The emergence of nosocomial infections with CoNS has illuminated the role of numerous virulence factors. In the context of infections and breaches of the skin barrier, these factors allow virulent adherence, persistence, and multiplication of CoNS. The use of whole-genome sequence data has evidenced the multiplicity of such factors in CoNS. It would be more appropriate to characterize these elements as pathogenicity factors, even if some of them can appear exceptionally as virulence factors that may lead to specific clinical conditions, as seen with *S. epidermidis*-producing strains.

The increasing data that are now available at the clinical, molecular, and genomic levels even make possible the development of innovative approaches to characterize bacterial pathogenicity. Deneke et al. proposed such a novel approach by using a machine learning workflow that determines the pathogenicity of a novel bacterial species based on genomic data only, an eventuality that is not rare with the increasing use of metagenomic analyses in various environments and microbiota [86].

Nevertheless, if sequencing technologies have become cheaper and whole-genome sequence data easier to produce, they will remain as complementary tools to molecular and clinical studies that have to be coordinated in a structured framework [87]. Good practices and quality controls have become crucial to ensure the quality of the released genomes and the validity of analyses [88,89].

## Figures and Tables

**Table 1:** *Staphylococcus epidermidis* whole-genome-based studies to determine virulence determinants.

Date	Ref	Type of Analysis	Genomes	Strain ID	Accession Number	Strain Origin	Putative Genetic Determinants of Pathogenicity	
**2003**	[40]	Pathogenicity factor search	1	ATCC 12228	NZ_CP022247	Skin swab	pSE-12228-01 pSE-12228-02 pSE-12228-03 pSE-12228-04 pSE-12228-05 pSE-12228-06	Plasmids
							*sar, agr*	Regulation systems
							*ssp*	Serine protease
							*geh*	Lipase
							*clp, sep*	Clp protease, metalloprotease, nuclease
							*hld*	Delta haemolysin
							*atl*	Autolysin
							*sdr, embp, ebps*	Adhesins/MSCRAMMs
2005	[41]	Pathogenicity factor search	1	RP62a	NC_002976.3	Catheter-associated infection	*clp, ssp, nuc*	Clp protease, serine and cysteine protease, nuclease
							*lip, geh*	Lipases
							*hlb, hld*	Haemolysins
							*psm*	Phenol-soluble modulins
							*sdr, ses, ebh, ebp, fbe*	Adhesins/MSCRAMMs
							νSEγ	Genomic island carrying PSMs genes
							νSE1, νSE2	Integrated plasmids carrying cadmium resistance gene and putative MSCRAMMs
							*cap*	Capsule synthesis
		Comparative genomics with 4 *S. aureus* genomes	2	Complete genomes from the study and from GenBank	-	-	-	*S. epidermidis* and *S. aureus* present syntenic genomes and horizontal gene exchange probably happen between those species in both directions, possibly mobilizing virulence factors from *S. aureus* to *S. epidermidis*.
2012	[42]	Comparative genomics between commensal and pathogenic *S. epidermidis*	30	Complete genomes from the study	-	-	-	*S. epidermidis* has an open pan genome, the variable genome comprises mobile genetic elements, the *fdh* gene distinguishes commensal from pathogenic bacteria.
2013	[43]	Pathogenicity factor search	1	12142587	GCA_000304575.1	Endocarditis	*ica*	Intercellular adhesion factors
							*fbe, sdr, ebp, ebh*	Adhesins/MSCRAMMs
							*dtl*, *mpr, vra, apr*	*Resistance to antimicrobial peptides*
							*ssp*	Serine and cysteine protease
							*lip, geh*	Lipases
							*psmβ*	Phenol-soluble modulin
							*hlb*	Beta-haemolysin
2015	[29]	Comparative genomics with 241 *S. aureus* genomes	83	Complete genomes from GenBank	-	-	-	*S. epidermidis* and *S. aureus* share a core genome of 1478 genes, and if homologous recombinations are rare between both species, interspecies transfer of mobile genetic elements might happen and shape the genome of both species regarding the environmental conditions.
2017	[30]	Comparative genomics between *S. epidermidis* strains after osteoarticular infections	104	Complete genomes from the study	-	-	-	Some genes from *S. epidermidis* were associated with bad outcome in patients: *bhp* (biofilm formation), *qacA* (antiseptics resistance), *ccrA-ccrB* (cassette chromosome recombinase), IS*256*-like (transposase).
2018	[44]	Comparative genomics between 3 *S. epidermidis* genomes	3	Complete genomes from the study and from GenBank	-	-	-	*S. epidermidis* pathogenic strains that produce a C-3 like enterotoxin bear a SEC-coding sequence located on a composite pathogenicity island *SePI-1/SeCI-1* that suggest the existence of HGT from *S. aureus* to *S. epidermidis.*
2018	[45]	Pathogenicity factor search	1	G6_2	ERR387168	Environmental strain	P1 to P6	Plasmids
							-	Presence of multiple genes implicated in drug resistance
							*atl*	Autolysin
							*ebh, ebp, sdr*	Adhesins/MSCRAMMs
							*ssp, nuc*	Serine and cysteine protease, nuclease
							*lip, geh*	Lipases
							*ica*	Intercellular adhesion factors
							*hlb, hld*	Haemolysin

**Table 2:** *Staphylococcus lugdunensis* whole-genome-based studies to determine virulence determinants.

Date	Ref	Type of Analysis	Genomes	Strain ID	Accession Number	Strain Origin	Putative Genetic Determinants of Pathogenicity	
**2011**	[50]	Pathogenicity factor search	1	N920143	NC_017353.1	Human breast abscess	*isd*	Iron-regulated surface determinants
							*sls, vWbl, fbl*	MSCRAMMs
							*sst*	Iron-regulated ferric siderophore uptake system
							*agr*	Regulation system
							*ess*	6 toxin secretion system
							*ica*	Intercellular adhesion factors
							*cap*	Capsule synthesis
							*mprf/dlt*	Immune evasion
							*CRISPR*	Clustered regularly interspaced short palindromic repeats sequence
							φSL1	Prophage
2017	[51]	Mobile genetic elements search	7	C_33 VISLISI_21 VISLISI_22 VISLISI_25 VISLISI_27 VISLISI_33 VISLISI_37	NZ_CP020768.1 NZ_CP020762.1 NZ_CP020764.1 CP020763.1 NZ_CP020735.1 NZ_CP020769.1 CP020761.1	Skin swab Bacteremia Endocarditis Knee Prosthesis Knee prosthesis Liver abscess Endocarditis	pVISLISI_1 to pVISLISI_5	Plasmids
							φSL2 to φSL4	Prophages
2018	[32]	Comparative genomics with 15 *S. aureus* and 13 *S. epidermidis* genomes	15	All complete genome from GenBank	-	-	-	Identification of a closed pan genome in *S. lugdunensis* and multiple barriers to horizontal gene transfer (restriction-modification, CRISPR/Cas, and toxin/antitoxin systems).

**Table 3:** *Staphylococcus capitis* whole-genome-based studies to determine virulence determinants.

Date	Ref	Type of Analysis	Genomes	Strain ID	Accession Number	Strain Origin	Putative Genetic Determinants of Pathogenicity	
**2015**	[46]	Pathogenicity factor search	1	AYP1020	NZ_CP007601.1	Bacteremia	pAYP1020	Plasmid
							-	Prophage
							-	Insertion sequence
							*agr, sar, sae, arl, rot, sigB*	Regulation system
							*ica*	Intercellular adhesion factors
							*cap*	Capsule synthesis
							*hlb*	Haemolysin
							*clp, ssp, sep, htr, spl*	Clp protease, cysteine and serine proteases, metalloprotease
							*lip, geh*	Lipase
							*psm*	Phenol-soluble modulins
							*fbe, aap, ebh, ses, ebp, bhp, sdr*	Adhesins/MSCRAMMs
							*atl*	Autolysin
2014	[47]	Drug resistance genes	1	LNZR-1	NZ_JGYJ00000000.1	Bacteremia	-	Presence of multiple genes implicated in drug resistance
2016	[48]	Pathogenicity factor search	4	NRCS-A CR01 CR03 CR04 CR05	NZ_CBUB000000000.1 NZ_CVUF00000000.1 NZ_CTEM00000000.1 NZ_CTEO00000000.1	Bacteremia	-	Plasmids
							IS256, IS272, IS431mec-like	Insertion sequences
							*hsdMSR*	Type Restriction/Modification system
							*-*	Presence of multiple genes implicated in drug resistance
							*atl*	Autolysin
							*ebh, ebp, sdr*	Adhesins/MSCRAMMs
							*ica*	Intercellular adhesion factors
							*clp, ssp, nuc*	Clp protease, cysteine and serine protease, nuclease
							*cap*	Capsule synthesis
							*hlb, hld*	Haemolysin
							*psm*	Phenol-soluble modulins
							*sar, rot, mgr*	Regulation system
2017	[49]	Pathogenicity factor search	1	TE8	NZ_JMGB00000000.1	Cutaneous swab	*Epidermicin-like* and *Gallidermin* gene clusters	Antimicrobial peptides
							*psm*	Phenol-soluble modulins with antimicrobial activities

**Table 4:** *Staphylococcus caprae, haemolyticus,* and *saprophyticus* whole-genome-based studies to determine virulence determinants.

Date	Ref	Type of Analysis	Genomes	Strain ID	Genome Accession number	Strain Origin	Putative Genetic Determinants of Pathogenicity	
*S. capare*								
2018	[54]	Pathogenicity factor search	3	JMUB145 JMUB590 JMUB898	AP018585 AP018586 AP018587	Bacteremia Nasal swab Bacteremia	*cap*	Capsule synthesis
							*clp, esp*	Clp protease
							*psm*	Phenol-soluble modulins
							*ses, bhp, sdr, clfB-like, fbe, embp*	Adhesins/MSCRAMMs
							*geh, lip*	Lipases
							*atl*	Autolysin
*S. haemolyticus*								
2005	[52]	Pathogenicity factor search	1	JCSC1435	NC_007168.1	Human	pSHaeA pSHaeB	Plasmids
							φSh1	Prophages
							*-*	Presence of multiple genes implicated in drug resistance
							*sdr, ebp*	Adhesins/SCRAMMs
							*spl, clp, nuc*	Clp protease, serine and cysteine proteases, nuclease
							*cap*	Capsule synthesis
							*atl*	Autolysin
							*hla*	Alpha-haemolysin
2016	[53]	Pathogenicity factor search	1	S167	NZ_CP013911.1	-	pSH108	Plasmid
							*agr, sar, arl*	Regulation systems
							*atl*	Autolysin
*S. saprophyticus*								
2005	[55]	Pathogenicity factor search	1	ATCC15305	NC_007350.1	Urine	pSSP1 pSSP2	Plasmids
							*pro, put, opu, aqp, nha*	Transport system related to urine environement
							*cap*	Capsule synthesis
							*uafA*	Adhesin specialized in urinary tract cells adherence
							*ure*	Urease activity
